# Scanning Tunneling Microscopy for Molecules: Manipulating Electron Transport through the Conduction Gap by Varying the Buffer Layer

**DOI:** 10.1021/acsphyschemau.5c00049

**Published:** 2025-09-10

**Authors:** Abhishek Grewal, Christopher C. Leon, Olle Gunnarsson

**Affiliations:** Max-Planck-Institut Für Festkörperforschung, Heisenbergstraße 1, Stuttgart 70569, Germany

**Keywords:** scanning tunneling microscopy, decoupling layer, buffer lattice parameter, molecular luminescence manipulation, alkali halides, up-conversion

## Abstract

In scanning tunneling microscopy of molecules, an insulating buffer layer is often introduced to reduce interactions between adsorbed molecules and the substrate. We demonstrate that the buffer itself strongly influences the wave function of the tunneling electron at the molecule for tunneling through the molecule’s electronic transport gap. We use a theory, which provides a very good agreement with prior experiments on platinum phthalocyanine on a NaCl buffer, to study effects of varying the buffer’s composition and thickness and show the importance of the buffer’s lattice parameter. Expanding the tunneling electron’s wave function using molecular orbitals (MOs) additionally shows how to control the relative weights at the highest occupied MO (HOMO), lowest unoccupied MO (LUMO), and energetically low-lying MOs with few nodal surfaces. Those with significant weight are key for manipulating molecules with tunneling electrons. When used for up-conversion molecular luminescence, in which emitted photons exceed the input tunneling bias, we find that an intricate competition occurs between tunneling through the HOMO or LUMO versus low-lying MOs. The buffer choice provides a substantive handle for controlling such processes. We predict that it can influence the up-conversion efficiency by an order of magnitude.

## Graphical abstract

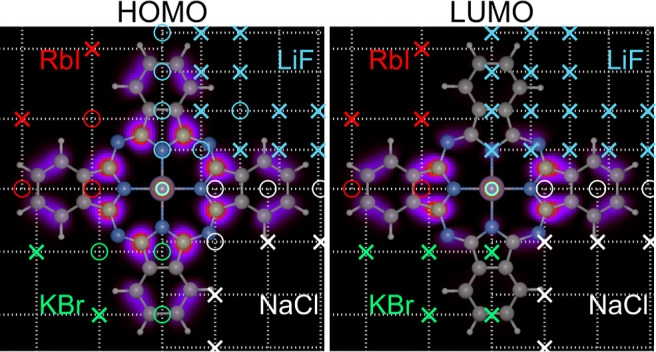

## Introduction

Scanning tunneling microscopy (STM) has been extensively used to study the intrinsic electronic structure of adsorbed molecules.^1–9^ In these studies, an atomically thin insulator such as a few monolayers of sodium chloride (NaCl) acts as a buffer between the substrate and the molecule.^10^ This buffer controls^11^ and reduces the influence of the underlying substrate on the molecule. It is commonly assumed to otherwise have just a small impact on STM, except for strongly reducing the total tunneling current. However, although the (indirect) coupling between the molecular orbitals (MOs) and the substrate is strongly reduced, we show that this reduction is very different for different MOs. This observation prompts focused interrogation of the buffer’s role in fundamental STM processes.

For instance, STM can induce molecules to emit photons. Experimental studies of single free-base phthalocyanine and platinum phthalocyanine (PtPc) molecules have shown up-conversion electroluminescence^12–15^ in which tunneling electrons cause the emission of photons with energy exceeding the applied bias voltage. One electron is believed to excite a triplet exciton in the molecule. A subsequent electron excites the triplet into a singlet exciton, which can then decay by emitting a photon. Negative differential resistivity is another interesting property seen in CuPc,^16–19^ where the total tunneling current is reduced as the bias is increased. In these cases, electrons often tunnel nonresonantly through the molecular electronic transport gap, making the process more complex than resonant tunneling.

Our efforts focus on how the buffer affects the tunneling through the transport gap. In addition to an exponential damping, we show that the buffer actually strongly influences the character of the wave function. Its presence in the STM junction cannot and should not be neglected. Together with effects from electron propagation through vacuum, this combination explains the dramatic changes of STM imaging when the bias is moved into the conduction gap. Of particular interest are the important changes taking place at the adsorbed molecule, which are shown to strongly depend on the buffer composition and thickness. An important consequence is that the choice of buffer expands the means of controlling the tunneling process and the manipulating of electrons and photons, in addition to the choice of adsorbed molecule. This can be used, e.g., to influence up-conversion electroluminescence or negative differential resistivity.

In the spatial range of an adsorbed molecule, the wave function of a tunneling electron can be approximated as a linear combination of molecular orbitals (MOs).^20,21^ These MOs couple to the substrate via the buffer states. For a complicated molecule, such as (planar) PtPc treated here, the highest occupied MO (HOMO) and energetically nearby orbitals, like the lowest unoccupied MO (LUMO), tend to have many nodal surfaces because they have to be orthogonal to many lower-lying MOs. We show that these Frontier orbitals then tend to couple inefficiently to the substrate. Low-lying (π) MOs, which have few or even no nodal surfaces perpendicular to the buffer surface, can have better effective coupling to the substrate and be orders of magnitude stronger than the HOMO coupling. This leads to extremely intricate and counterintuitive tunneling mechanisms.

Although these low-lying MOs are energetically far from an electron tunneling through the transport gap, strongly reducing their relative importance, they still play a significant role in describing the tunneling electron wave function,^20,21^ even at energies that are only slightly off resonance from the HOMO or LUMO for the reasons discussed below. This sensitivity enables varying the buffer composition and thickness to tune the relative importance of different MOs in the tunneling process. It is important to re-emphasize these effects as they have profound experimental and theoretical implications.

Heuristically, the p_*z*_ orbitals of the buffer, pointing out of the plane of the buffer toward the molecule, may be expected to play the most important role for the coupling, since the p_*x*_ and p_*y*_ orbitals point along the plane. The s orbitals would then be expected to be intermediate between these two extreme cases. However, the buffer atoms close to the adsorption site of PtPc typically sit at very symmetric positions relative to PtPc, as illustrated in Figure 1. From symmetry arguments, it then follows that the s and p_*z*_ orbitals on these sites, marked by “o” in the middle panel, cannot couple to the HOMO. Counterintuitively, only s and p_*z*_ orbitals relatively far from the adsorption site, marked by “x”, as well as some p_*x*_ and p_*y*_ orbitals, couple. Similar arguments apply also to several other MOs with many nodal surfaces. Given these circumstances, what possibilities exist for relatively strong coupling? Consider the lowest PtPc π MO (left panel of Figure 1), which has no nodal surface perpendicular to the buffer surface and therefore couples also to central s and p_*z*_ buffer orbitals. This leads to a very efficient coupling to this MO and to other MOs with few nodal surfaces. The buffer, therefore, has a drastic effect on the relative coupling to different MOs by an electron tunneling through the gap and thereby on the character of the tunneling electron.

**Figure 1:**
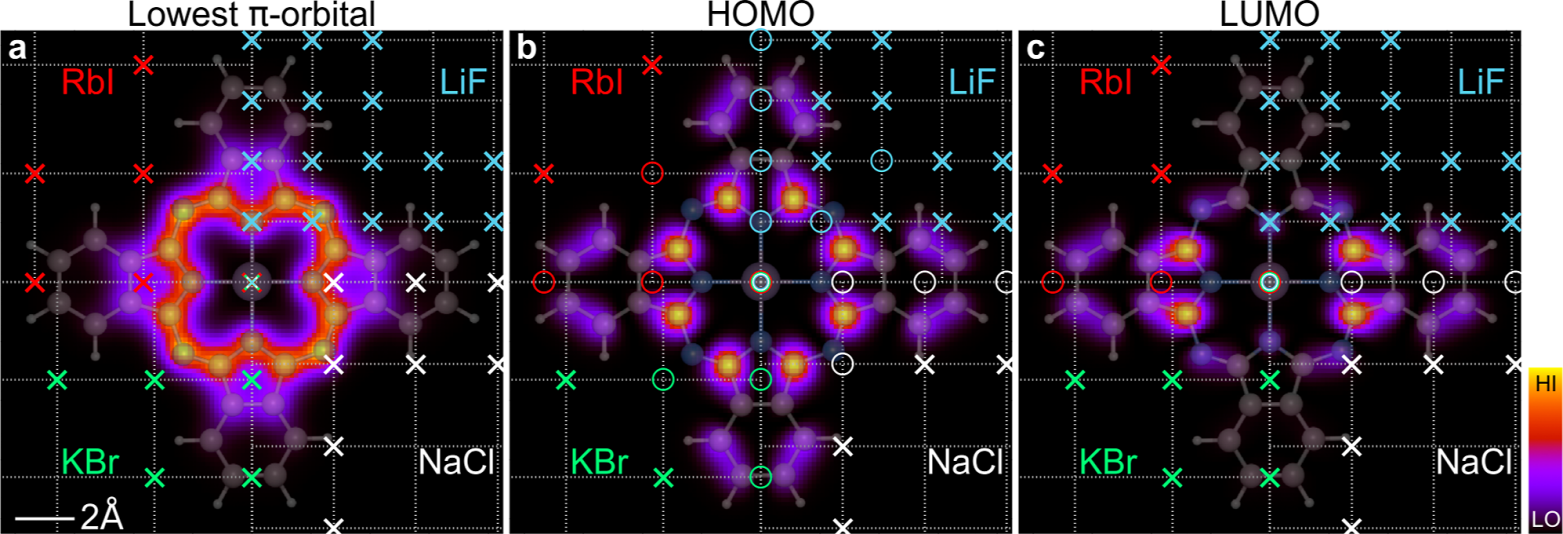
Norm squared of the PtPc molecule’s lowest π orbital (left), HOMO (middle) and LUMO (right) in a plane between the PtPc molecule and the buffer (at *z* = −1 Å). The symbols mark the underlying buffer lattice positions for the LiF (upper right), NaCl (lower right), KBr (lower left) and RbI (upper left) buffers, emphasizing the small number of RbI sites close to the molecule. The Pt atom is assumed to be adsorbed on top of a cation. The symbol “o” marks positions for which the buffer s and p_*z*_ orbitals do not couple to the PtPc MO for symmetry reasons, while “x” marks positions where coupling is possible. Because of the many nodal planes of the HOMO, it does not couple to s- and p_*z*_-orbitals at sites with high symmetry relative to the HOMO. Due to the large lattice parameter of RbI, these sites are essentially outside the range of the molecule, while for LiF a few such sites are not.

The discussion above refers to the coupling of a PtPc MO to a specific orbital on a specific buffer atom. For the coupling to a buffer eigenfunction there are additionally interference effects between the couplings to different buffer atomic orbitals. However, summing over buffer eigenfunctions, these interference effects tend to cancel out. The arguments above therefore address dominating effects.

This raises a question of how the chemical composition of the buffer influences this coupling. We consider several different buffers, namely LiF, MgO, NaF, NaCl, KBr, and RbI, all simple salts. Due to the very different sizes of the ions involved, the lattice parameters span almost a factor of 2, from 4.02 Å (LiF), 4.21 Å (MgO), 4.63 Å (NaF), 5.54 Å (NaCl), 6.59 Å (KBr) to 7.34 Å (RbI). This means that for LiF and MgO, ions in nonsymmetric positions relative to PtPc are closer to the center of the molecule and therefore couple more efficiently to MOs with many nodes than, e.g., RbI. The choice of buffer, therefore, has a large influence on the character of the wave function of electrons tunneling through the gap and its coupling to different MOs. This discussion is based on symmetry arguments and the geometry of system, and it is independent of the calculation method.

An interesting application is up-conversion, as alluded to earlier. Chen et al.^12^ showed that this happens also for a single adsorbed molecule, e.g., H_2_Pc, thereby excluding many earlier proposed mechanisms requiring interactions between two molecules. Their mechanism for a positive bias larger than the LUMO energy is shown in Figure 2. Up-conversion has been observed for both negative (H_2_Pc on an Ag substrate) and positive (H_2_Pc or PtPc on an Au substrate) bias.^12,13,15^ We show that the efficiency of these processes should depend substantially on the choice of buffer.

**Figure 2:**
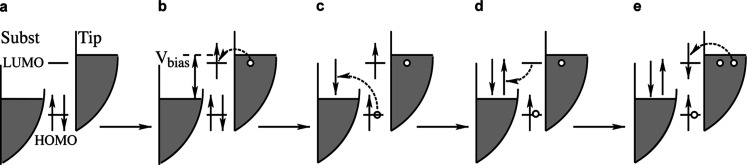
Singlet creation in a four-step process for *E*_triplet_ < *E*_LUMO_ < *V*_bias_ < *E*_singlet_. Figures a-c show the creation of a triplet due to the transfer of a tip electron to the LUMO and a HOMO electron to the substrate and d-e show how the triplet is converted into a singlet by the transfer of a second electron via the LUMO.

The case when the value of the (positive) bias is smaller than the LUMO energy is particularly interesting. Detailed comparisons of theoretical and experimental images show very good agreement and illustrate that tunneling still mainly occurs via MOs.^21^ Contrary to what is sometimes stated, an exciton can still be formed, in an overall energy-conserving many-body process, via tunneling through the HOMO and LUMO, as above. The process is now, however, much less efficient. There are then important competing processes where an electron tunnels from a low-lying MO to the substrate and subsequently a tip electron tunnels to the emptied MO, without creating an exciton. By varying the buffer we can strongly influence the relative importance of such competing processes. We also briefly discuss processes involving tunneling through low-lying levels combined with a Coulomb induced transition also leading to an exciton and up-conversion.

We use a tight-binding (TB) formalism, taking into account the substrate (Au, Ag, or Cu), the buffer (LiF, MgO, NaF, NaCl, KBr, or RbI), and the 182 bound orbitals of the PtPc molecule. This provides a suitable starting point for later many-body treatments, and it shows the origin of the surprising results obtained below. The corresponding formalism is described in Supporting Information. The manner of electron propagation from the substrate to the molecule is the primary focus of this paper. The propagation through vacuum was discussed extensively in ref 21, as well as in its accompanying Supporting Information. The calculations here follow a similar approach.

We have demonstrated that this TB formalism yields results in very good agreement with experiment. This applies both to the description of the HOMO and LUMO as well as the images within the conduction gap.^21^ Additionally, we have shown that the formalism appropriately describes images for different orientations of the adsorbed molecule, and have discussed the effects of different adsorption sites.^20^

## Methods

### Theoretical Formalism

We study a (planar) PtPc molecule adsorbed on a few layers of a buffer atop a noble metal substrate. As earlier,^20–22^ we use a TB formalism to describe the substrate-buffer-PtPc molecule complex, essentially following prescriptions of Harrison^23^ (see Supporting Information). We write the corresponding Hamiltonian as

1 $$\begin{array}{l} H=\sum_{i\sigma }\varepsilon_{i}^{Sub}n_{i\sigma }^{Sub}+\sum_{i\sigma }\varepsilon_{i}^{Buff}n_{i\sigma }^{Buff}+\sum_{i=1}^{182}\sum_{\sigma }\varepsilon_{i}^{PtPc}n_{i\sigma }^{PtPc}\\ +\sum_{ij\sigma }\left[V_{ij}^{Sub‐Buff}{\left(c_{i\sigma }^{Sub}\right)}^{†}c_{j\sigma }^{Buff}+h.c.\right]\\ +\sum_{ij\sigma }\left[V_{ij}^{Buff‐PtPc}{\left(c_{i\sigma }^{Buff}\right)}^{†}c_{j\sigma }^{PtPc}+h.c.\right] \end{array}$$

where h.c. is the complex conjugate. The first three terms yield the energies of the substrate states, the buffer states, and the 182 PtPc states, respectively. The last two terms describe the hopping between the substrate and the buffer and between buffer and PtPc, respectively. It has been shown that the conduction band of NaCl is mainly located on the Cl 4s level.^22,24–26^ Here we assume that the conduction band is located on the anion also for the other buffers.

In the Harrison method the only parameter entering the hopping integrals between s and p orbitals on two neighboring atoms is the separation of the two atoms. The orbital energies only depend on the atom on which the orbitals are centered. As discussed in the Supporting Information, the Pt spin–orbit coupling is neglected, since it has a small effect on the π-orbitals important for the PtPc coupling to the substrate and the tip. The values of the various parameters are discussed in Supporting Information. We use the Cu(111), Au(111) and Ag(100) substrate surfaces and neglect possible reconstructions. In all cases, we assume that PtPc adsorbs on the cation site with the same orientation as it has on NaCl on Au (see Supporting Information). Considering the absence of relevant experimental information in the literature, it is the simplest assumption to make which lets us focus on the great importance of the buffer lattice parameter. For a given real system, there may be appreciable additional effects due to the specifics of adsorption processes that occur on it, see, e.g., ref 20.

The Hamiltonian in eq 1 is used for describing the system inside a matching plane at *z*_0_ = 1 Å outside the nuclei of the PtPc molecule. Outside this plane, we introduce cylindrical coordinates with the radial coordinate, ρ, the azimuthal angle, ϕ, and a coordinate perpendicular to the surface, *z*, see Supporting Information and ref 21. We neglect changes of the potential induced by the tip. We use the theory of Tersoff and Hamann^27,28^ to describe the tunneling interaction with the tip. With these assumptions, the tip does not enter the calculations explicitly. This approach was used to construct the images in Figures 3 and 9 below.

**Figure 3:**
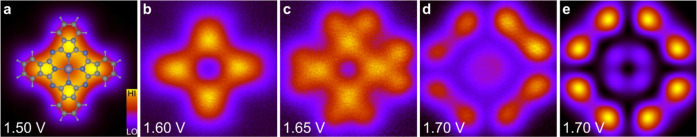
Constant height experimental STM images (b–d) at different biases illustrating the sharp transition when the bias is increased from within the transport gap (at 1.60 and 1.65 V) to the LUMO (1.70 V). The leftmost and rightmost panels (a,e) show theoretical results for 1.50 and 1.70 eV, respectively. All panels show an area of 20 × 20 Å^2^. Panels d and e illustrate that the theory accurately describes the LUMO (1.70 V). As the bias is lowered below the LUMO the image changes very rapidly and dramatically. For 1.70 V the there is very little weight along the *x*- and *y*-axes while for 1.6 V most of the weight is along these axes. This is well described by theory (see panel a), only including propagation through MOs and neglecting propagation directly from the buffer to the tip. The overlaid PtPc molecule in panel a clearly shows that the apparent STM image when propagating through the gap is smaller than the molecule.

Neglecting the attractive potential from the substrate-buffer system for *z* > *z*_0_ as well as the neglect of the potential from the tip tends to overestimate the rapid decay of the wave functions in the vacuum region. The HOMO and LUMO positions are adjusted to the values determined from STM for PtPc on NaCl on Au.

As long as the important PtPc states are the neutral ground-state and states with one extra electron or one hole, Coulomb effects and image potential effects are implicitly included by the use of level positions determined by STM. Below the notations HOMO and LUMO refer to these effective level positions. Exciton effects are not included, except in some of the qualitative discussions of up-conversion below.

For other combinations of buffer and substrate, the HOMO and LUMO alignments may be substantially different, but in most cases, we are not aware of corresponding experimental results. We have therefore used the same alignments as for NaCl on Au, focusing on the effects of the buffer composition and thickness.

The Hamiltonian (eq 1) is expressed in terms of atomic orbitals (AO) and we express the solutions of the Hamiltonian as linear combinations of these AOs. This leads to a matrix formulation and the solutions are found by direct diagonalization of the Hamiltonian matrix. From these solutions the results in Figures 4 and 5 below were obtained. On the PtPc molecule, an eigenstate |*i*⟩ of the full system is described as a linear combination of the 182 eigenstates |ν⟩ of the free PtPc molecule isolated in the gas phase

2 $$|i\rangle =\sum_{\nu =1}^{182}c_{\nu }^{\left(i\right)}|\nu \rangle$$

We can then define a partial density of states on the PtPc molecule

3 $$N_{\nu }\left(\varepsilon \right)=\sum_{i}{\left|c_{\nu }^{\left(i\right)}\right|}^{2}\delta \left(\varepsilon -\varepsilon_{i}\right)$$

and a partial occupancy

4 $$w_{\nu }=C\int_{\varepsilon_{1}}^{\varepsilon_{2}}N_{\nu }\left(\varepsilon \right)\;d\varepsilon$$

The gap of the PtPc model extends from −1.3 to 1.7 eV. We choose ε_1_ = – 0.8 eV and ε_2_ = 1.2 eV, which is purposely a little bit inside the gap. The prefactor was chosen so that the sum over the 182 weights *w*_ν_ add up to unity, that is

5 $$C^{-1}=\sum_{\nu }\int_{\varepsilon_{1}}^{\varepsilon_{2}}N_{\nu }\left(\varepsilon \right)\;d\varepsilon$$

**Figure 4:**
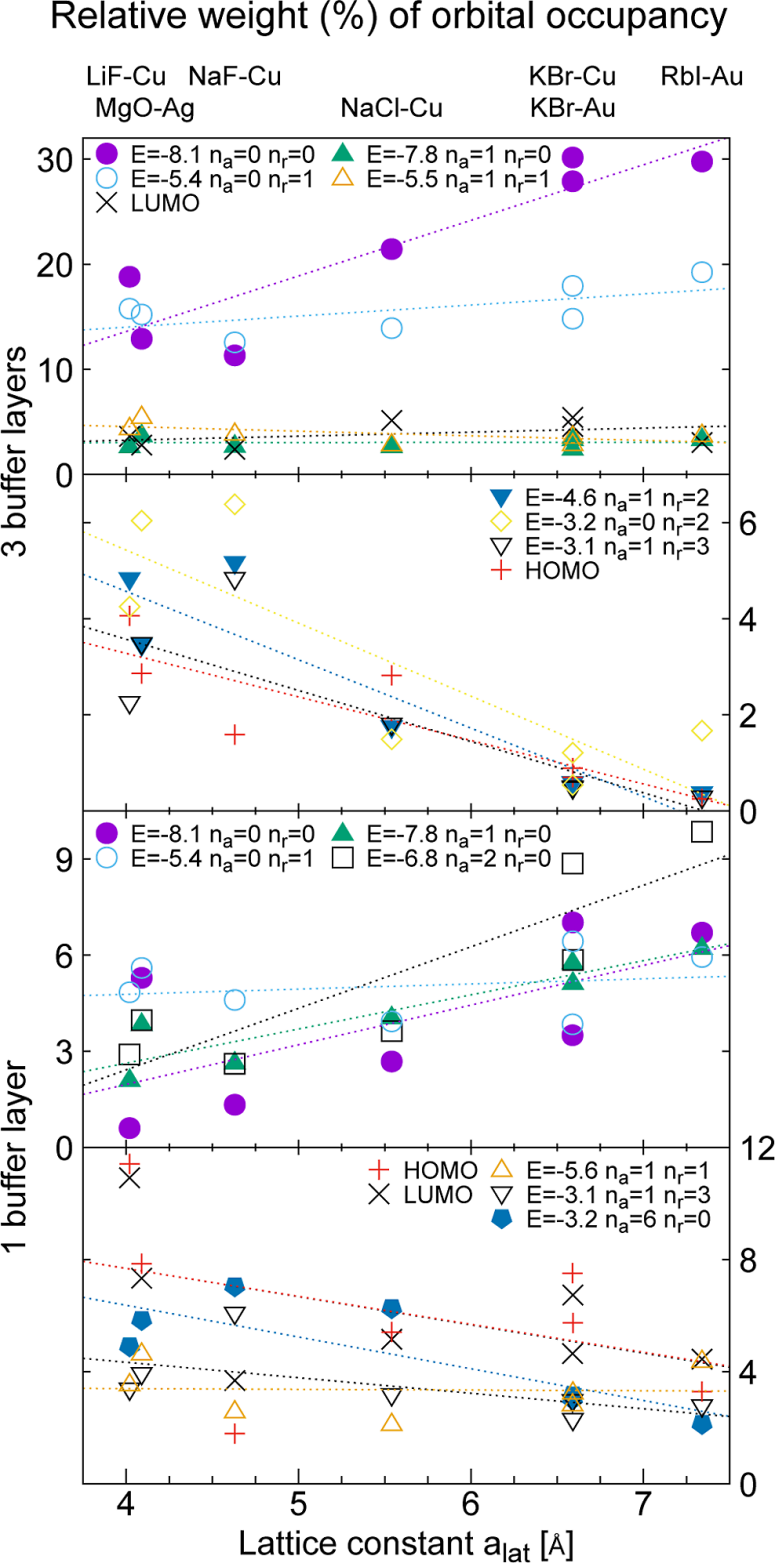
Partial occupancy *w*_μ_ for important PtPc MOs for three (top two panels) and one (bottom two panels) buffer layers integrated over the energy interval −0.8 to 1.2 eV. We consider the buffers LiF, MgO, NaF, NaCl, KBr, and RbI on substrates Cu, Ag, or Au, and plot the results as a function of the buffer lattice parameter. The MOs are labeled by the number of angular nodes, *n*_a_, and radial nodes, *n*_r_. Dotted straight lines have been fitted to the results for a given MO. Observe that the vertical scale is a factor ≈2.7–4.6 larger for the top panel than the lower three panels. The lines are guides for the eye.

**Figure 5:**
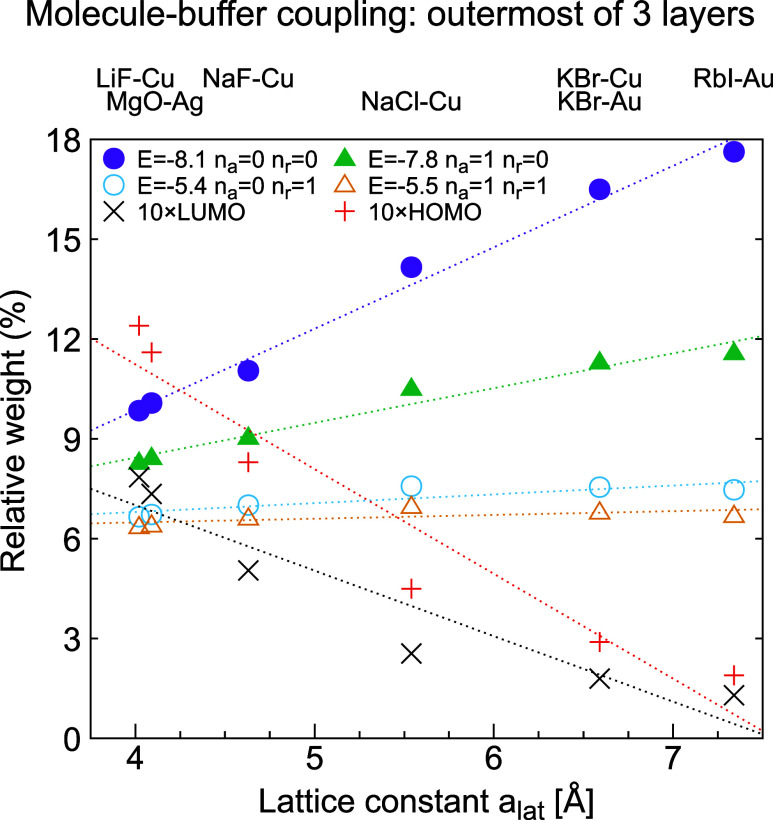
Coupling, *A*_ν_, of MOs to outermost buffer layer (eq 7) for different buffers as a function of the buffer lattice parameter *a*_lat_ and for the case of three buffer layers. Results are shown for a few low-lying MOs with few nodal surfaces and strong coupling and for the HOMO and LUMO. Observe that the coupling strengths to the HOMO and LUMO are multiplied by a factor 10, illustrating the weak coupling to these orbitals. For the 2-fold degenerate orbitals we show the coupling to one of the orbitals. The dotted straight lines are guides for the eye.

We now focus on the MOs built up from p_*z*_ orbitals on atoms in the molecule. The p_*z*_ orbitals point toward both the tip and the underlying buffer, and the corresponding MOs are therefore particularly important for the current. Some of these MOs can be classified according to the number of angular nodal surfaces, *n*_a_, and radial nodal surfaces, *n*_r_. Here *n*_a_ refers to the number of nodal surfaces which are (approximately) planes through the center of the molecule and perpendicular to the plane of the molecule. In a similar way, *n*_r_ refers to nodal surfaces which are approximately cylinders perpendicular to the plane of the molecule. There are also other orbitals that fall outside of this method of classification.

It is sometimes suggested that for a bias in the transport gap, electrons propagate directly from the buffer to the tip, outside of the adsorbed molecule. We neglect this effect, based on calculations along the lines of refs 20 and 21 , shown in Figure 3. The figure shows that theory describes the image accurately, both when the bias is at the LUMO, as well as the very different image when the bias is slightly below the LUMO. In particular, the image slightly shrinks, both experimentally and theoretically, when the bias is lowered. If the electrons would propagate outside the molecule, we would expect the image to expand and to leave a large hole in the middle, contrary to experiment. The analysis of the calculation shows that the off resonance image is dominated by low-lying π-orbitals well below the HOMO and LUMO (ref 21), which are favored exponentially over the HOMO or LUMO during propagation from the molecule to the tip.

## Results and Discussion

Figure 4 shows the weights of important orbitals for energies, −0.8 ≤ ε ≤ 1.2 eV, inside the PtPc energy gap for PtPc on a three (top two panels) or one (bottom two panels) layer atomic buffer for different combinations of substrate and buffer. For degenerate orbitals, we show the weight of one orbital. The coupling to the substrate orbitals has a substantial dependence on the specific energy interval chosen, but the integrated quantities give the main trends. Below we discuss the energy dependence in more detail.

We first consider three buffer layers (two top panels). In the top panel the weights of the orbitals increase, stay constant or moderately decrease with the buffer lattice parameter, while in the second panel the weights rapidly decrease. In the upper panel the number of nodal surfaces is typically small. In particular, the two orbitals with no angular node (*n*_a_ = 0) and with zero (*n*_r_ = 0) or one radial node (*n*_r_ = 1) have large relative weights, which increase with the buffer lattice parameter. In the second panel the weights of the orbitals rapidly drop with the lattice parameter and become very small for the largest lattice parameters (but do note the change in vertical scale from the top figure). These orbitals typically have more nodal surfaces. The LUMO orbital, with several nodal planes, has a rather weak lattice parameter dependence in Figure 4. This is a peculiarity of the energy interval studied. Figure 6 below shows that the LUMO has a substantial energy and lattice parameter dependence.

**Figure 6:**
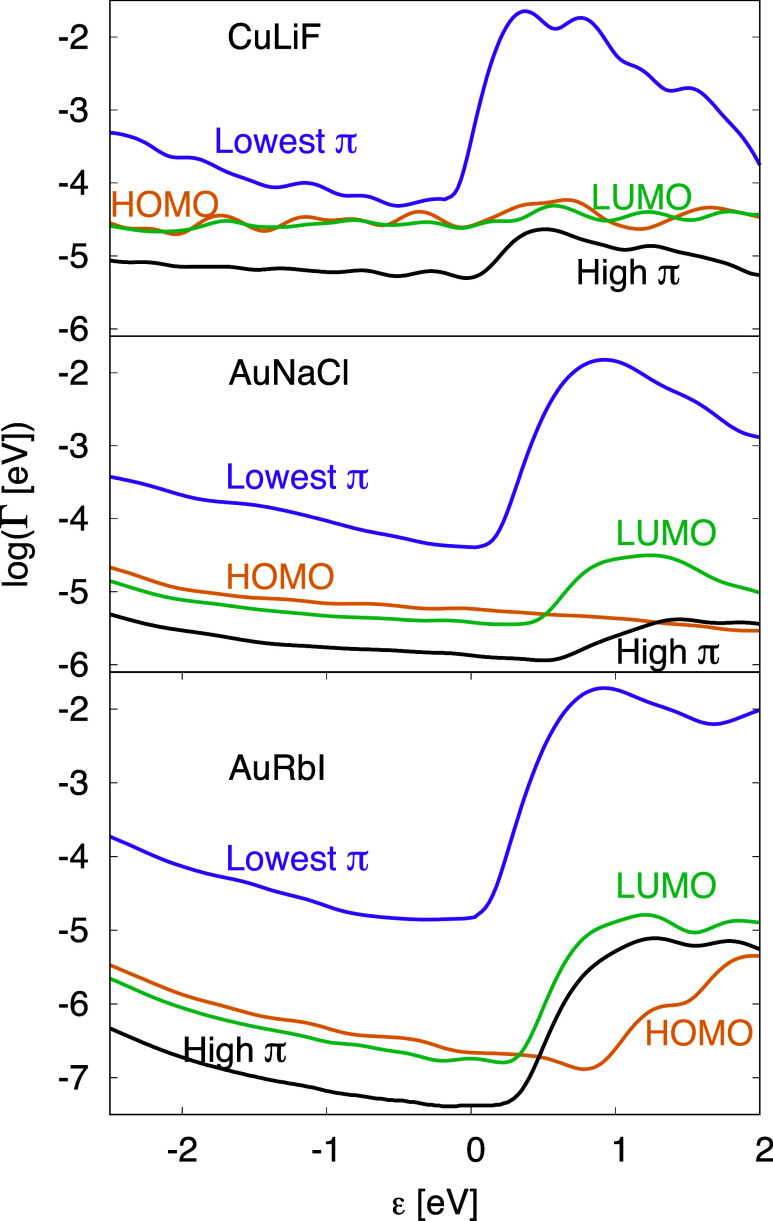
Coupling log­(Γ_ν_) [eq 8] between an MO ν and the substrate-buffer system as a function of the energy ε of the coupling substrate states. Results are shown for the CuLiF, AuNaCl and AuRbI systems coupling to the HOMO, LUMO, the lowest π-state and a π-state about 5 eV above the LUMO.

We next consider one monolayer thick buffer (two bottom panels). In the third (fourth) panel we show MOs for which the weights tend to increase (decrease) with the lattice parameter of the buffer. As for the case of three layers, the weights of MOs with few (many) nodal surfaces tend to increase (decrease) as the lattice parameter increases. Thus, orbitals with few and many nodal surfaces typically behave in qualitatively different ways for both one and three buffer layers.

In perturbation theory, we might expect the weight of a MO to be given by

6 $$\sum_{-0.8\leq \varepsilon_{n}\leq 1.2}{\left|\sum_{i}\frac{\langle Substn|H|Buffi\rangle \langle Buffi|H|\nu \rangle }{\left(\varepsilon_{i}-\varepsilon_{n}\right)\left(\varepsilon_{\nu }-\varepsilon_{n}\right)}\right|}^{2}$$

where ε_ν_ is the energy of the PtPc νth MO, ε_*i*_ is the energy of a buffer state *i* and ε_*n*_ the energy of a substrate state. The second energy denominator in eq 6 is then in the range 0.5–2.5 eV for the HOMO or LUMO but about 8 eV for the lowest π orbital, i.e., about an order of magnitude larger. The energy denominator then favors the HOMO or LUMO by about 2 orders of magnitude. Nevertheless, Figure 4 shows that the weight of the lowest π orbital is larger and will be discussed in the next section.

These results here refer to the weights in the plane of the molecule for the different MOs for an electron tunneling through the transport gap. In ref 21 we focused on the propagation through vacuum outside the molecule, continuing the MOs into vacuum. It was shown that this propagation, in addition, exponentially favors some components of the wave function already strongly favored in Figure 4, e.g., the lowest π orbital.

### Effects of the Nodal Structure of Molecular Orbitals

We now discuss why different MOs have very different effective coupling to the substrate. Figure 1 shows the lowest π orbital, the HOMO, and one of the two degenerate LUMOs in a plane between the PtPc molecule and the buffer. In addition, the figure shows the sites of the LiF (upper right), NaCl (lower right), KBr (lower left) and RbI (upper left corner) buffer layer. Based on these figures, we now discuss the strength of the coupling of the three MOs to different buffers. One may expect the coupling of PtPc to the buffer s and p_*z*_ orbitals to dominate over the coupling to the p_*x*_ and p_*y*_ orbitals, which point along the buffer surface. We, therefore, focus on the former set of orbitals.

Let us first consider the lowest π orbital, which has no nodes except in the plane of the molecule. This orbital then couples to all s and p_*z*_ orbitals of the buffer within the spatial extension of the molecule. Because of the larger lattice parameter of, e.g., RbI than, e.g., LiF, there is coupling to fewer buffer orbitals for RbI, even though there is still a substantial coupling also for RbI.

We next consider the HOMO. Because of the many nodal planes perpendicular to the buffer, the HOMO does not couple to the s and p_*z*_ orbitals on many sites in symmetric positions relative to PtPc. These sites are marked by “0” (see Figure 1). In particular, this applies to all positions close to the center. Orbitals on sites further out are in nonsymmetric positions and can couple. However, only a few of these orbitals are spatially within the extension of the molecule, and they mainly couple to the outer parts of the HOMO. This is particularly important for buffers with a large lattice parameter. Figure 1 illustrates that for LiF there are still a few such buffer sites inside the PtPc molecule, whereas for RbI these sites are really only in contact with the outer edges of the PtPc. Due to the efficient suppression of the coupling to buffer s and p_*z*_ orbitals, the coupling to p_*x*_ and p_*y*_ orbitals becomes relatively more important.

Each of the LUMO orbitals has a node along one of the coordinate axes, and therefore does not couple to s or p_*z*_ orbitals on that axis. The sites for which s and p_*z*_ orbitals can couple are then rather far out, particularly for RbI. These sites have weak couplings to the LUMOs, in a similar manner to the HOMO.

To illustrate these effects, we focus on the hopping matrix elements between a MO ν and a buffer basis function *i*. The sum of the squares of these matrix elements is then calculated as

7 $$A_{\nu }=\sum_{i}\left|\langle Buffi|H\right|\nu \rangle {|}^{2}$$

The results for *A*_ν_ are shown in Figure 5 for different buffers. The figure illustrates how the couplings to the HOMO and LUMO are strongly reduced relative to the coupling to several low-lying π orbitals, especially as the lattice parameter of the buffer, *a*_lat_, is increased. For this reason the HOMO and LUMO have rather small weight in Figure 5, although they are greatly favored by their energetic closeness [small energy denominators in eq 6] to the energy interval studied.

### Coupling between Different MOs and Substrate States

The results in, e.g., Figure 4 are based on diagonalization of the full Hamiltonian in eq 1 and integrated over a finite energy range. To obtain a better understanding of the results, we now proceed in two steps. First the combined substrate-buffer part of the Hamiltonian [eq 1] is diagonalized, providing solutions |*a*⟩ with energies ε_*a*_. Then the molecular part is diagonalized, giving solutions |ν⟩ with the energy ε_ν_. Finally we calculate the coupling, *V*_ν*a*_, between these two groups of solutions. In the qualitative discussion we neglect the indirect coupling between the MOs via the buffer and substrate. This then leads to the Anderson impurity model.^29^ The coupling as a function of energy is given by

8 $$\Gamma_{\nu }\left(\varepsilon \right)=\pi \sum_{a}|V_{\nu a}{|}^{2}\delta \left(\varepsilon -\varepsilon_{a}\right)$$

Γ_ν_(ε) is shown for PtPc adsorbed on LiF (substrate Cu), on NaCl (Au) and RbI (Au) in Figure 6. We show results for the HOMO, LUMO, the lowest π MO at about 8 eV below *E*_F_ and a π MO at about 5 eV above *E*_F_. The figure illustrates how the coupling to the lowest π-orbital is very strong, one to 3 orders of magnitude larger than the coupling to the HOMO and LUMO, and with a strong energy-dependence. The HOMO and LUMO have different energy dependencies for buffers with a larger lattice parameter, with the LUMO coupling, in particular, to higher states in the PtPc transport gap. The high-lying π-orbital shown in Figure 6 has an atypically strong coupling for such a high-lying orbital, not much weaker than the HOMO and LUMO.

We next study these couplings for different buffers and for the energies ε = 0.4 eV (HOMO) and 1.6 eV (LUMO, lowest π-state and a high π-state). As we shall show below, results for these energies are essential for up-conversion at positive bias. Figure 7 shows that the HOMO coupling is substantially weakened compared with the LUMO coupling as the buffer lattice parameter (*a*_lat_) is increased. This is essential for up-conversion, as discussed below.

**Figure 7:**
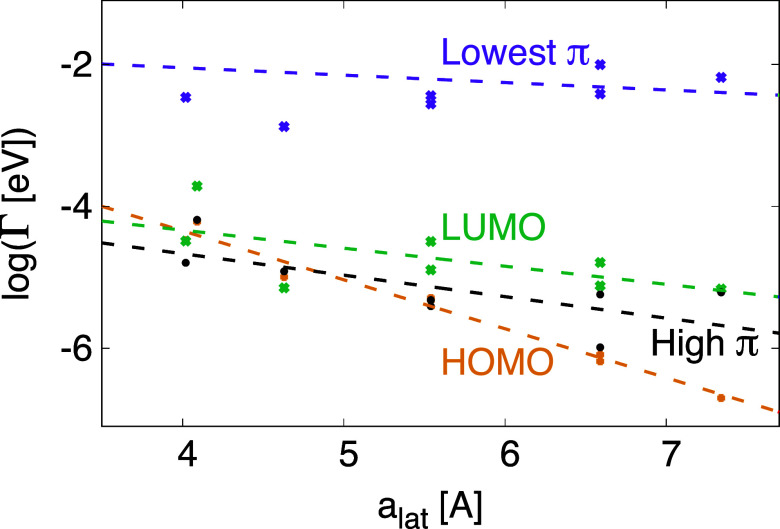
Coupling log­(Γ_ν_(ε)) between the substrate and the HOMO, LUMO, lowest π-state and a π-state about 5 eV above the HOMO as a function of the lattice parameter of the buffers (LiF, MgO, NaF, NaCl, KBr and RbI). For the HOMO the coupling was calculated for substrate states at ε = 0.4 eV and in the other three cases for ε = 1.6 eV.

### Exciton Formation and Up-Conversion

We now consider up-conversion and photon emission. The full treatment requires a many-body theory, but our one-particle results provide important input. The case of a positive bias, larger than the LUMO energy, was described in Figure 2 in the introduction. In step *a* → *b* a tip electron tunnels to the LUMO. In a following step *b* → *c* a HOMO electron tunnels to a substrate state, at approximately 0.4 eV, in the presence of a LUMO electron, creating a triplet exciton.^15^

In a competing process (not shown in the figure), the LUMO electron can tunnel to a substrate state, at approximately 1.6 eV, giving no contribution to triplet formation and luminescence. The relative probability for these two processes is very important for the efficiency of up-conversion. Figure 7 shows the coupling strength for different levels as a function of the buffer lattice parameter. Tunneling of the HOMO electron, giving a triplet, is greatly disfavored by a large buffer lattice parameter, e.g., a RbI buffer, while for a LiF buffer the HOMO and LUMO coupling strengths are comparable. Figure 7 suggests more than an order of magnitude difference between LiF and RbI, with NaCl being intermediate. The following step *c* → *d* is efficient compared with competing processes, since the LUMO to substrate tunneling is efficient compared with tip to molecule tunneling for typical tip distances. In the final step *d* → *e* there is a competing process where the HOMO hole is filled by a tip electron, again resulting in no exciton. This competition should have a weak buffer dependence. Overall, there is then a strong buffer dependence in exciton formation due to the *b* → *c* step. We observe that the lowest π MO may not play an important role when the bias is larger than the LUMO energy, in spite of the strong coupling, since the processes discussed above are very efficient.

We next consider the case when *E*_triplet_ < *V*_bias_ < *E*_LUMO_. It has been implied that the sequence in Figure 2 is then forbidden by energy conservation, since the tip Fermi energy is now below the LUMO, and that the step *a* → *b* in Figure 2 would not be possible.^12,15^ The full process *a* → *e* is, however, also in this case emphatically allowed, provided that the initial state *a* and the final state *e* are degenerate.^20^ This can be seen in, e.g., perturbation theory.

Using a detailed comparison of theory and experiment, we have shown that tunneling via MOs is not only allowed but very important for the (weak) tunneling through the conduction gap.^21^ In particular, we showed that tunneling through low-lying π MOs is very important. For instance, lowering the bias by just 0.1 V below the LUMO completely changes the experimental image. This drastic change is very well reproduced by theory. Theory shows that even for energies very close to the LUMO, tunneling occurs primarily via MOs rather far below the LUMO in energy, in particular via the lowest π MOs. Actually, the image is fairly similar to this orbital. This explains the drastic change of the image as the bias is lowered and shows the importance of tunneling through such MOs.^21^

This illustrates that even in the conduction gap, tunneling is primarily inside the molecule, not outside. Tunneling directly from the buffer to the tip through the vacuum outside the molecule should give a very different image (see also the discussion in the context of Figure 3).

These results illustrate that the creation of an exciton via tunneling through the HOMO and LUMO is possible also for *E*_triplet_ < *V*_bias_ < *E*_LUMO_. However, it also illustrates that tunneling via low-lying π-orbitals, without creating an exciton, is a very important competition.

We now discuss various possible processes in the context of H_2_Pc on an Ag surface, for which up-conversion happens for negative bias. In this case the HOMO is at about −2.3 eV. If the bias is more negative than −2.3 V a singlet can simply be created by a HOMO electron tunneling to the tip and a substrate electron tunneling to the LUMO. This can then be followed by photon emission.

The more interesting case is when *E*_singlet_ < |*V*_bias_| < |*E*_HOMO_|. The same two-step process is still allowed, although now less efficient, but probably still very important. Interestingly, we notice an additional mechanism, illustrated in Figure 8a. By invoking a Coulomb induced transition that occurs among the PtPc electrons it is possible to create a HOMO–LUMO exciton in H_2_Pc without relying on the inefficient tunneling to and from the HOMO and LUMO. The corresponding Coulomb integral can be large, of the order of one eV (see Supporting Information). The corresponding tunneling matrix elements are then, in addition, very large, one to 2 orders of magnitude larger than for the HOMO and LUMO. Such processes could give a non-negligible contribution to up-conversion.

**Figure 8:**
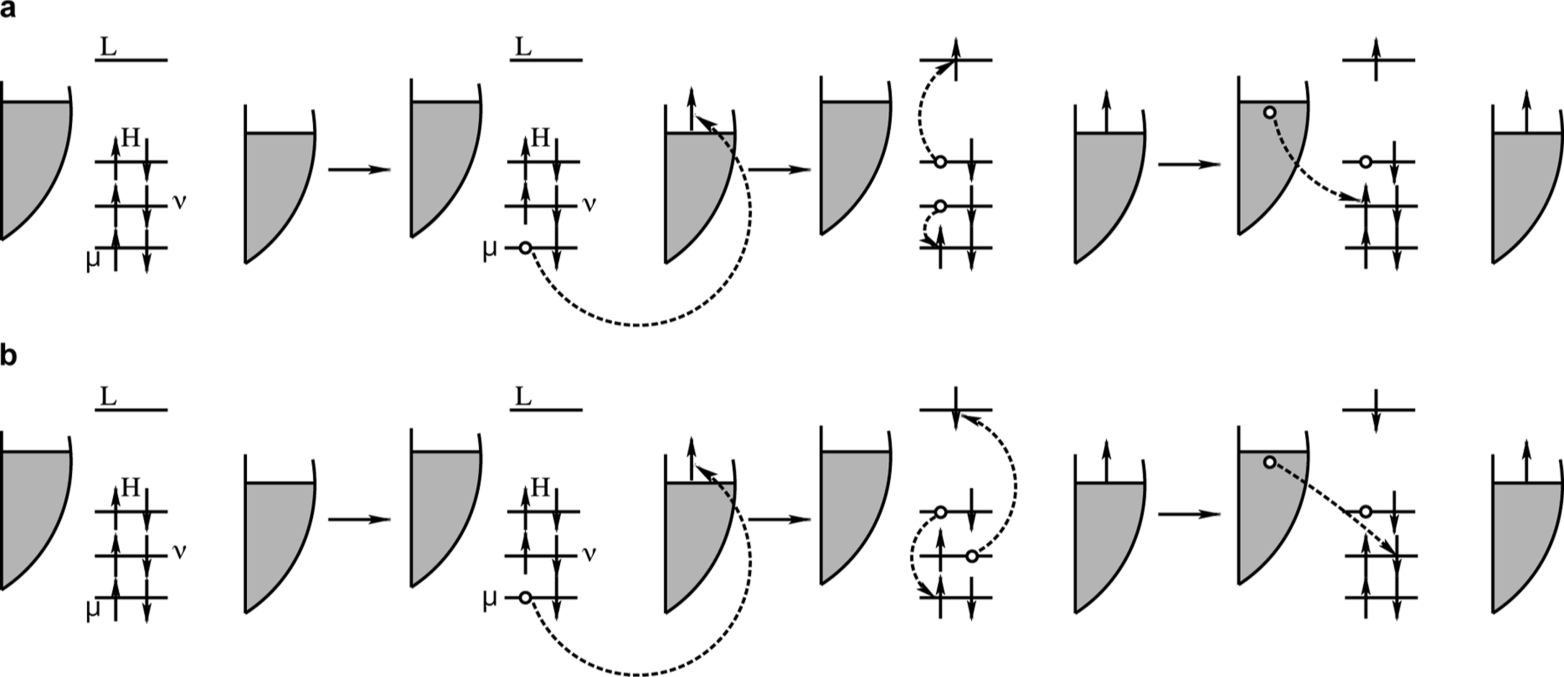
Processes leading to the creation of an exciton without tunneling to or from the HOMO or LUMO, of interest for the case when the bias is in the conduction gap. In (a) a singlet is created and in (b) a triplet. In (a) the electrons tunneling between the substrate and the molecule and between the molecule and the tip have the same spins, while in (b) they have different spins. In a first step an electron tunnels from a low-lying MO to the tip. In (a), in a Coulomb induced transition, the HOMO electron is then excited into the LUMO and an electron in a low-lying level fills the hole created in the first step. Finally the just created hole is filled by a substrate electron. In (b), in a Coulomb induced transition, the HOMO electron falls down into the hole created in the previous process and another electron is excited into the LUMO. The hole just created is then filled by a substrate electron.

If *V*_bias_ < |*E*_singlet_| a singlet cannot be created in a two-step process. Instead, a four-step process is needed. This could then be achieved in a similar way as for positive bias by two tunneling electrons, first creating a triplet exciton and then a singlet exciton. The energetics is such that the latter process is very efficient, while the former is the crucial process.

In contrast to the two-step process above the net spin of the molecule has to be changed to create a triplet. This can easily be achieved if the electrons tunneling from the substrate to the molecule and from the molecule to the tip have different spins. No (probably very inefficient) spin flip mechanism needs to be introduced and only tunneling processes to and from the molecule are needed.

In similar processes as above, the intermediate triplet exciton can also be created by tunneling through low-lying MOs followed by a Coulomb induced transition. In this case, however, there are stronger constraints on the Coulomb induced transitions (see Figure 8b). We estimate these Coulomb integrals to be of the order of 0.1 eV. The quantitative importance of the processes in Figure 8 is not yet clear.

In an alternative mechanism, where an electron tunnels directly from the buffer to the tip in vacuum outside the molecule, a spin-flip process would be required to create the intermediate triplet state of the molecule. This could happen due to, e.g., spin–orbit coupling. Such a process should be highly inefficient, however, both because the spin–orbit coupling of the light atoms in, e.g., H_2_Pc is very weak and because this would require a spin–orbit induced interaction between electrons in essentially different parts of space.

The relative probability for different processes can be influenced by the choice of buffer, since different tunneling matrix elements have large dependencies on the buffer lattice parameter (see Figure 4). This opens up a rich scenario of possibilities for manipulating the up-conversion process in a desirable way.

Above we have discussed the case of a positive bias for an Au substrate and a negative bias for an Ag substrate. In Supporting Information we show that an Ag substrate is unfavorable for a positive bias and an Au substrate for a negative bias. The energies involved for the four cases are discussed in more detail in Supporting Information.

### Effects of Vacuum Propagation for Tunneling through the Transport Gap

Up to this point, we have discussed tunneling through the transport gap, mainly focusing on the different components of the wave function at the adsorbed molecule. The focus was on how this influences, e.g., up-conversion. We now shift our focus to the image seen when the tip is scanned over the molecule. Figure 9 shows results for the two buffers with the smallest and largest lattice parameter (LiF and RbI) and for two thicknesses, one or three layers.

**Figure 9:**
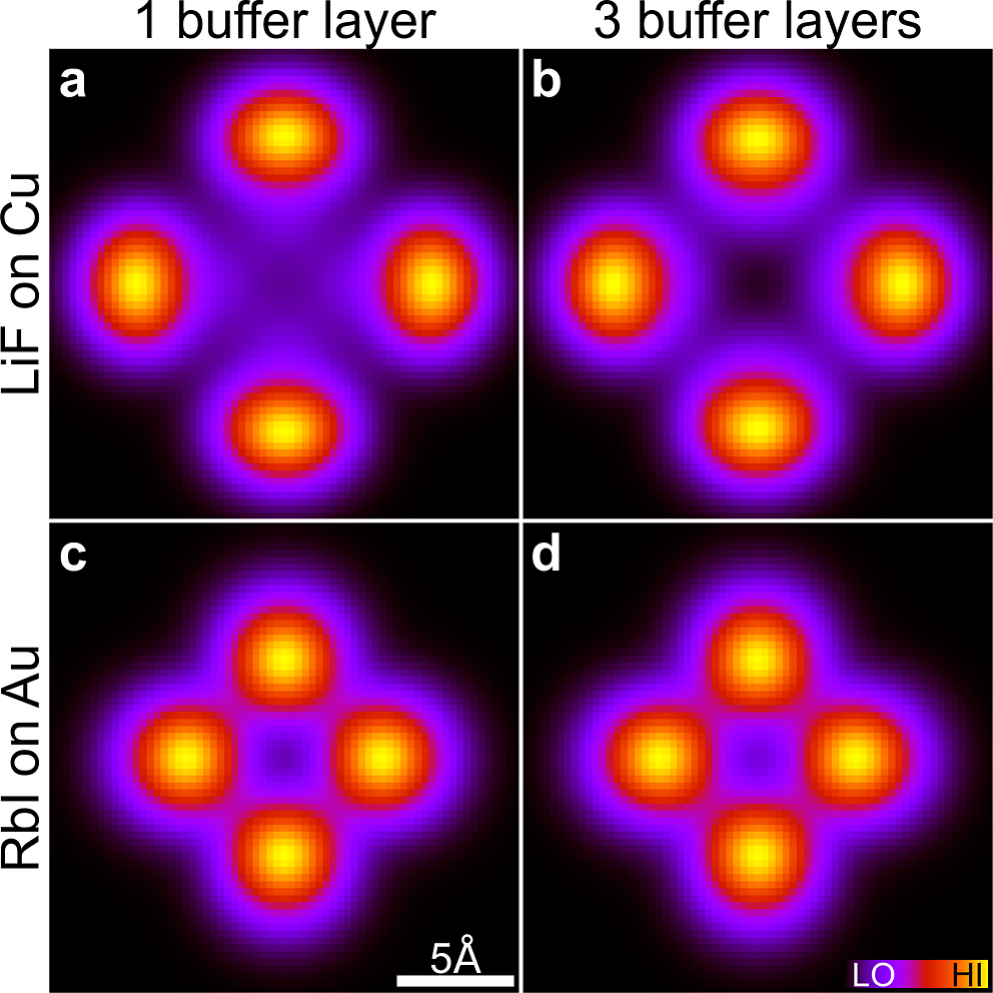
Images at *z* – *z*_0_ = 6 Å and ε = 0 for PtPc on LiF on Cu (top row) and on RbI on Au (second row) for a buffer with one layer (left) or three layers (right). All panels show an area of 20 × 20 Å^2^.

It is striking that the differences are rather small. A pattern with a somewhat wider spacing is found for LiF, although it has a smaller lattice parameter. It is interesting that the spatial range of the image does not follow the lattice parameter of the buffer but, rather, is determined by the specific coupling. The small difference between images for very different buffers might suggest the traditional picture, where the buffer just isolates the molecule from the substrate but otherwise does not influence the image much. However, this is a common misinterpretation.

We demonstrated earlier that the weight of some components at the molecule depends very strongly on the buffer. For instance, the weight of the HOMO varies by 1 order of magnitude between the different buffers. However, the weight of components with a more complicated nodal structure, such as the HOMO, have rather small weights already at the adsorbed molecule. These components are further strongly (exponentially) damped during propagation through vacuum relative to, e.g., the lowest π orbital.^21^ The image at the tip of an electron tunneling through the transport gap is, therefore, dominated by components with very simple nodal structures,^20,21^ primarily components with no or one angular node. Components with more complicated angular structures play just a very small role. The strong relative variation of the weights of these more complicated orbitals with the buffer lattice parameter then has a barely noticeable effect on the image at the tip, independently of the buffer.

## Conclusions

We have studied the tunneling through the transport gap of a molecule, PtPc, focusing on effects of a buffer between the molecule and the substrate. We studied the wave function of the tunneling electron in the spatial region of the molecule, and expressed it as a linear combination of MOs of the molecule.

We demonstrated that the transport through the buffer greatly favors π-MOs with a simple nodal structure compared with the HOMO, LUMO and other MOs with complex nodal structures. The complex nodal structure follows from the requirement that the HOMO and LUMO are orthogonal to 22 energetically lower π-orbitals. It leads to a poor coupling, via the buffer, to the substrate. Despite the HOMO and LUMO being hugely favored by their proximity in energy to the tunneling electron, MOs at substantially lower energies tend to dominate the wave function of an electron tunneling through the transport gap.

The lattice parameter of the buffer plays a crucial role, since a large buffer lattice parameter, in particular, disfavors MOs with a complex nodal structure. For instance, the relative weight of the HOMO in the transport gap is suppressed by 1 order of magnitude in going from a LiF to a RbI buffer. Additionally, we found that the number of atomic layers of the buffer also plays a role.

During the propagation through vacuum, the components of the wave function corresponding to a few low-lying π orbitals, already favored by the buffer, are further exponentially favored by vacuum propagation. Just a couple of components dominate and the image in the transport gap becomes relatively independent of the buffer (see Figure 9).

For applications involving manipulation of electrons, such as formation of HOMO–LUMO excitons, up-conversion, photon emission and negative differential resistivity, the electron wave function in the spatial region of the molecule is very important. In this region additional components of the wave function can play an appreciable role for an appropriate choice of buffer (see Figure 4). The change of the relative weight of the HOMO by an order of magnitude then becomes important. This should be a useful tool for controlling many-body processes in the molecule. Traditionally, these applications relied on an appropriate choice of an adsorbed molecule. The results above, however, demonstrate two additional possibilities for influencing these processes - the choice of the buffer chemical composition, particularly the lattice parameter, and the number of buffer atomic layers.

The tunneling of electrons in the conduction gap through low-lying MOs is an important competition to the direct formation of HOMO–LUMO excitons. If Coulomb induced transitions in the molecule are taken into account, however, a HOMO–LUMO exciton can also be created with the help of such tunneling processes. A HOMO–LUMO exciton is then created without involving the (inefficient) tunneling to or from the HOMO or LUMO. This raises interesting questions about the relative importance of such processes, which can be influenced by the choice of buffer.

## Supplementary Material



## Data Availability

The data supporting this study’s findings are available from the corresponding authors upon reasonable request.

## Notes

Tight-binding model, substrates, buffers, model of PtPc, vacuum propagation, exciton formation (PDF)

## †

Département de chimie, Université Laval, 1045 avenue de la Médecine, Québec QC, G1V 0A6, Canada
