# Supplementary material for: Scanning Tunneling Microscopy for Molecules: Manipulating Electron Transport through the Conduction Gap by Varying the Buffer Layer
Source: ACS Phys Chem Au. 2025 Sep 10;5(6):599–608. doi: 10.1021/acsphyschemau.5c00049 (PMC12670310; doi:10.1021/acsphyschemau.5c00049)
Supplement: Supplementary file 1 [file pg5c00049_si_001.pdf]

Scanning Tunneling Microscopy for Molecules: Manipulating Electron Transport through the Conduction Gap by varying the Buffer Layer

Abhishek Grewal,[*,†] Christopher C. Leon,[*,†,‡] and Olle Gunnarsson[*,†]

†*Max-Planck-Institut für Festkörperforschung, Heisenbergstraße 1, 70569 Stuttgart, Germany*

‡*Present address: Département de chimie, Université Laval, Québec, Canada*

E-mail: a.grewal@fkf.mpg.de; christopher.leon@chm.ulaval.ca; o.gunnarsson@fkf.mpg.de

Supplementary Information

We describe the tight-binding models for PtPc on different buffers on a Cu, Ag, or Au substrate, generalizing earlier descriptions for NaCl on Au[1,2] to the new substrates and buffers. We generally use the tight-binding formalism of Harrison[3] to obtain nearest-neighbor hopping integrals between basis functions located on the atoms.

The buffer and substrate are incommensurate in all cases except MgO on Ag. While the nearest neighbors are well-defined for hopping within the substrate, the buffer, and PtPc, they are ill-defined for hopping between the substrate and the buffer and between the buffer and PtPc. We, therefore, use a smooth distance-dependent cutoff of the Harrison prescription for the two latter cases. The Harrison hopping integrals are multiplied by a factor

$$e^{-(d-d_i)^2/\lambda_i^2}, \quad i = \text{B or M}, \tag{1}$$

for these hoppings.

For the substrate-buffer hopping B, d is the distance between a substrate atom and a buffer atom at the substrate-buffer interface, and d_B is the separation between the adjacent substrate-buffer planes. The parameter λ_B is chosen so that, on average for each buffer atom, the factors in Eq. 1 add up to four, representing a typical number of nearest neighbors in a neighboring plane. For the buffer-PtPc hopping, d is the distance between a PtPc atom and a buffer atom in the outermost plane, and d_M is the separation between the outermost buffer plane and the plane of PtPc. Again, λ_M is chosen so that, on average, these factors add up to four for each PtPc atom.

The orbital energies are essentially obtained from Harrison.[3] However, we have modified some energies to correct known inaccuracies. Thus, we shift the energy of the substrate d orbital so that the distance of the top of the d-band to the Fermi energy agrees with results in the literature. Finally, we shift all states uniformly so that the Fermi energy $E_F = 0$.

We have shown that for NaCl the conduction band primarily has anion s character.[4] We assume that the corresponding results also apply to MgO and the other alkali halides. Thus, we adjust the level energies of the buffers so that the conduction band has primarily anion s, and the experimental gaps are reproduced. These level energies then also include, implicitly, Madelung

and image potential effects. Finally, we shift all levels uniformly, to obtain the correct location of the top of the valence band relative to E_F, when this location is known.

The parameters of PtPc are discussed below. The tight-binding wave functions are then matched to the exponentially decaying wave function in the vacuum region outside PtPc as described in Ref. 2.

Whenever experimental results are not known for some parameter, we use the parameters for NaCl on Au as a guide. This then puts the focus on known differences between the different systems, in particular, the different lattice parameters of the buffers.

Although we use models for the substrate with a large number of atoms, the detailed results depend on the precise arrangement of the atoms in the substrate. In most cases we then perform calculations with different number of layers and different number of atoms per layer, giving a total number of substrate atoms of around $3000 - 4500$. For the buffer we typically use 324 atoms per layer. This then leads to matrices of sizes up to about 45000×45000. The results of these different calculations are then averaged.

Substrates

We use the lattice parameters $a_{\mathrm{Cu}} = 3.60$ Å,[5] $a_{\mathrm{Ag}} = 4.09$ Å[6] and $a_{\mathrm{Au}} = 4.07$ Å.[5] The top of the d-band is put at 2.0 eV,[7] 4.0 eV[8] and 1.6 eV[9] below the Fermi energy for Cu, Ag, and Au, respectively. We use the Cu(111), Ag(100), and Au(111) surfaces. The s and d level energies were obtained from Harrison, and we added a p level about 5 eV above the s level. Finally, the Harrison parameters were slightly modified to obtain the desired d-band position relative to E_F.

Buffers

LiF

We use the lattice parameter 4.02 Å.[10] Starting from the known separation of the innermost KBr layer on Cu, 2.90 Å,[11] we estimate the corresponding distance for LiF on Cu by using Shannon's

ionic radii.[12] Assuming that the larger F^- ions determine the separation, we obtain the estimate $2.90 + 1.33 - 1.96 = 2.27$ Å. The band gap of LiF is 13.6 eV,[13] and the Li and F parameters were adjusted accordingly. Based on Ref. 14 we estimate that the top of the valence band of a thin film of LiF on Au is about 7 eV below the Au Fermi energy and use the same alignment for LiF on Cu.

MgO

The MgO lattice parameter is adjusted from 4.21 Å to the value for Ag,[6] $a_{\mathrm{MgO}} = a_{\mathrm{Ag}} = 4.09$ Å, to make the two lattices commensurate. We use the Ag-MgO separation $d_{\mathrm{Ag-MgO}} = 2.62$ Å.[6] O atoms are put on top of Ag atoms.[15] The Mg atoms then sit in hollow positions. After these adjustments, the density of O atoms and Ag atoms per layer is the same. The O and Mg levels are adjusted so that the bulk band gap of MgO is 7.8 eV.[16] All MgO levels are shifted so that the top of the O 2p band is 4 eV below E_F.[15]

NaF

We use the lattice parameter 4.63 Å.[10] The band gap of NaF is 11.5 eV.[17] Based on Shannon's ionic radii,[12] we reduce the distance of KBr on Cu 2.90 Å to $2.90 + 1.33 - 1.96 = 2.29$ Å. The band gap of NaF is 11.5 eV.[17] We put the top of the valence band at -6 eV, so that the substrate Fermi energy is approximately located in the middle of the gap.

NaCl

Calculations find that for a three layer NaCl film on Au the lattice parameter is reduced from the NaCl bulk value to 5.54 Å,[6] the value used here. We use the calculated separation $d_{\mathrm{Au-NaCl}} = 3.12$ Å between the Au surface and the NaCl film.[6] The band gap of NaCl is 8.5 eV.[18] Based on GW[19] calculations,[20] we put the top of the NaCl valence band 5 eV below the E_F.

KBr

For KBr we use the lattice parameter $a_{\mathrm{KBr}} = 6.60$ Å.[21] We use the calculated separation $d_{\mathrm{Cu-KBr}} = 2.90$ Å[11] between the KBr film and the Cu substrate. For KBr on Au we use the separation for KBr on Cu and add the difference in Cu and Au metallic radii[22] to obtain the separation $d_{\mathrm{Au-KBr}} = 3.06$ Å. The KBr parameters are adjusted so that the band gap is 7.6 eV.[23] Since we do not know the position of the KBr valence band with respect to E_F, we put the top of the band at 5 eV below E_F, as for NaCl on Au.

RbI

For RbI we use the lattice parameter 7.34 Å.[24] Based on the separation of NaCl from Au and the differences in ionic radii between Cl and I[12] we use the separation 3.51 Å between the RbI and Au planes. The band gap of RbI is 6.1 eV.[25] For lack of experimental results, we put the top of the valence band at 4 eV below E_F. This puts the top somewhat higher than for NaCl on Au. Due to the smaller gap for RbI we used this value to avoid that the gap is too asymmetric relative to the Fermi energy.

Model of PtPc

We study the absorbed PtPc molecule. The coordinates of PtPc are obtained from a density functional calculation. The tight-binding parameters are obtained from Harrison[3] and are given in Table 1. For the H atoms we include the 1s level at the energy -13.6 eV (not given by Harrison). We then slightly shifted the HOMO and LUMO for each substrate-buffer-PtPc combination so that the PtPc HOMO forms a resonance at -1.3 eV and the LUMO a resonance at 1.7 eV, in agreement with experiment for PtPc on NaCl on Au. Since we are not aware of experimental results for HOMO and LUMO positions in most of the other combinations of substrate and buffer, this alignment was used in all cases. Adjusting the HOMO and LUMO energies to experimentally observed positions means that various effects are implicitly included, e.g., charge rearrangement in PtPc in the presence of a LUMO electron or a HOMO hole, as well as polarization of the buffer

Table 1: Level energies used in the models. The top three lines give substrates parameters, the following 12 lines buffer parameters and the last four lines PtPc parameters.

Element	*s*	*p*	*d*
Cu (4s, 4p, 3d)	6.0	11.0	−5.7
Ag (5s, 5p, 4d)	4.1	9.0	−5.9
Au (6s, 6p, 5d)	4.1	9.1	−3.7
Li (2s,2p)	23.0	29.0	–
Na (NaF)(3s, 3p)	18.1	22.1	–
Na (NaCl)(3s, 3p)	12.8	16.8	–
Mg (3s, 3p)	19.5	23.8	–
K (4s, 4p)	10.0	14.0	–
Rb (5s, 5p)	8.5	12.5	–
O (3s, 2p)	17.1	−4.0	–
F (LiF)(3s, 2p)	20.2	−7.0	–
F (NaF)(3s, 2p)	15.5	−6.0	–
Cl (4s, 3p)	10.2	−5.0	–
Br (5s, 4p)	6.8	−5.0	–
I (6s, 5p)	5.3	−4.0	–
C (2s, 2p)	−19.38	−11.07	–
N (2s, 2p)	−26.22	13.84	–
Pt (6s, 5d)	−6.85	–	−16.47
H (1s)	−13.61	–	–

due to charging of the molecule

For PtPc on NaCl on Au,[1,2] we follow Miwa *et al.*[26] and use the separation 3.4 Å between the the molecule and the NaCl film, absorbed on top of a Na atom. The four arms of PtPc are along the NaCl (100) directions. For the hopping between the molecule and the buffer we use an exponential cut-off as described above so that, on average, each atom in PtPc hops to four atoms in the buffer. The Au slab breaks the four-fold symmetry of PtPc which has been reintroduced in the results.

We have neglected the spin-orbit coupling for the Pt 5*d* electrons. The PtPc π-electrons, playing the essential role in this work, only couple to two of the five Pt 5*d*-orbitals, namely the *xz* and *yz*

orbitals. Most of this coupling is exhausted by a two-fold degenerate π orbital about 10 eV below the LUMO and with about 87 % Pt 5d character. These two orbitals are then rather localized on Pt, well inside PtPc, and with little interaction with the substrate or the tip. Several other PtPc π-orbitals of particular interest here have no coupling to Pt 3d for symmetry reasons. This is true for, e.g., the HOMO and the lowest π-orbital, where the latter orbital is important because of its strong coupling to the substrate and the tip. In other cases the mixing of Pt 5d orbitals into PtPc π-orbitals is very weak. The main contributions to the LUMO come from basis states of the type sin(5ϕ), which do not couple to the Pt 5d orbitals. The LUMO only couples via a very small component sin(ϕ). Together with the large energy separation ($\sim$ 10 eV), the result is a very weak mixing of about 0.001 Pt 5d weight into the LUMO. Two-fold degenerate levels about 6 and 9 eV below the LUMO have larger, but still rather small, Pt 5d weights of about 8 and 5 %, respectively.

We are not aware of experiments for PtPc giving the separation to the buffer or the orientation of PtPc in most other cases. We then construct models which follow PtPc on NaCl on Au as much as possible. In particular, we assume that PtPc is absorbed on the positive ion and has the same orientation as for PtPc on NaCl on Au. However, based on Shannon's "ionic radii",[12] we modify the separation to the buffer according to the size of the anion in the buffer.

The difference in the results between the different cases will then be primarily due to the choice of buffer and substrate. Should later experiments show that PtPc is absorbed on a different site or has a different orientation, our calculations in Ref. 1 provide guidance about the corresponding changes.

Vacuum propagation

The tight-binding description above is used outwards to a distance $z_0 = 1$ Å above the molecule. For the description outside this plane we follow Ref. 2. For $z > z_0$, we assume a constant potential, V_0, inside a cylinder with radius $\rho_0 = 12$ Å, and infinite outside. We use the work

function $V_0 = 4.3$ eV[27] and the substrate Fermi energy is used as energy zero. Then

$$V(\rho,\phi,z) = \begin{cases} V_0, & \text{if } \rho \le \rho_0 \text{ and } z \ge z_0; \\ \infty & \text{if } \rho > \rho_0 \text{ and } z \ge z_0. \end{cases} \tag{2}$$

The cylinder radius (12 Å) is much larger than the distance from the cylinder axis to the outermost H atoms (7.6 Å). We introduce the Schrödinger equation for an energy $E(< V_0)$

$$\left[-\left(\frac{\partial^2}{\partial z^2} + \frac{1}{\rho}\frac{\partial}{\partial \rho} + \frac{\partial^2}{\partial \rho^2}\right) + \frac{1}{\rho^2}\frac{\partial^2}{\partial \phi^2} + V(\rho,\phi,z)\right]\psi(\rho,\phi,z) = E\psi(\rho,\phi,z) \tag{3}$$

where $m(\ge 0)$ is an integer and J_m is an integer Bessel function. The solution is given by

$$\psi(\rho,\phi,z) = \sum_{mi}\left[c^{(s)}_{mi}\sin(m\phi) + c^{(c)}_{mi}\cos(m\phi)\right] J_m\left[k_{mi}\rho\right] e^{-\kappa_{mi}z}, \tag{4}$$

where $m(\ge 0)$ is an integer and J_m is an integer Bessel function. The coefficients k_{mi} are defined so that $J_m[k_{mi}\rho_0] = 0$, to guarantee that the wave function is zero for $\rho = \rho_0$. To obtain the correct energy, E, we require

$$[\kappa_{mi}]^2 = [k_{mi}]^2 - (E - V_0). \tag{5}$$

All energies are expressed in Ryd=13.6 eV and lengths in Bohr radii $a_0 = 0.529$ Å. The functions $\sin(m\phi)$, $m \ge 1$ and $\cos(m\phi)$, $m \ge 0$ describe the dependence on the azimuthal angle ϕ. For a given value of m, the Bessel functions $J_m[k_{mi}\rho]$, $i = 1, 2, \ldots$ describe the radial behavior. Finally, $\exp(-\kappa_{mi}z)$ gives the exponential decay in the z-direction. These vacuum solutions are matched continuously to the tight-binding solutions for substrate-barrier-molecule complex. The resulting image is given a Gaussian broadening with the full width half maximum of 3.2 a_0 to simulate experimental resolution.

Coulomb integrals

In the discussion of exciton formation we introduced intramolecular Coulomb integrals inducing intramolecular transitions. These integrals are defined in terms of the molecular orbitals (MOs)

$$|i\rangle = \sum_{\nu=1}^{182} c_\nu^{(i)} |\nu\rangle, \tag{6}$$

where $|\nu\rangle$ stands for the 182 atomic orbitals of PtPc. The corresponding Coulomb integrals we write as

$$U_{ij,kl} = \sum_{\mu\mu'\nu\nu'} (c_\mu^{(i)})^* c_{\mu'}^{(j)} u_{\mu\mu',\nu\nu'} (c_\nu^{(k)})^* c_{\nu'}^{(l)} \tag{7}$$

We introduce the atomic orbital Coulomb integrals

$$u_{\mu\mu',\nu\nu'} = \int d^3r d^3r' \phi_\mu^*(\mathbf{r}) \phi_{\mu'}(\mathbf{r}) \frac{e^2}{|\mathbf{r}-\mathbf{r}'|} \phi_\nu^*(\mathbf{r}') \phi_{\nu'}(\mathbf{r}') \tag{8}$$

We make the approximation

$$u_{\mu\mu',\nu\nu'} = v_{\mu,\nu} \delta_{\mu\mu'} \delta_{\nu\nu'}, \tag{9}$$

i.e., we neglect the rather small Coulomb integrals involving factors of the type $\phi_\mu^*(\mathbf{r})\phi_{\mu'}(\mathbf{r})$, where $\phi_\mu(\mathbf{r})$ and $\phi_{\mu'}(\mathbf{r})$ are orthogonal orbitals if $\mu \neq \mu'$. Furthermore we use

$$v_{\mu,\nu} = \begin{cases} v_0 & \text{for } \mu = \nu \\ \frac{e^2}{|\mathbf{R}_\mu - \mathbf{R}_\nu|} & \text{for } \mu \neq \nu \end{cases} \tag{10}$$

Here $\mathbf{R}_\mu$ are the positions of the atoms in the PtPc molecule. We use $v_0 = 12$ eV.

Up-conversion for different substrates and polarities

In the main text we discussed HOMO-LUMO up-conversion for, e.g., H_2Pc on a NaCl buffer on an Au substrate for positive bias and for an Ag substrate for negative bias. We now show that an Ag substrate is unfavorable for up-conversion for a positive bias and an Au substrate for negative

Table 2: The HOMO and LUMO energies for H_2Pc on NaCl on an Ag or Au substrate, and the triplet and singlet exciton energies.[28,29] The energy zero is the substrate Fermi energy.

	Ag	Au
E_{HOMO} (eV)	-2.25	-1.1
E_{LUMO} (eV)	0.9	1.6
E_{Trip} (eV)	1.2	1.2
$E_{\mathrm{Sing.}}$ (eV)	1.81,1.92	1.81,1.92

bias. Some relevant energies are shown in Table 2. Here we only consider tunneling through the HOMO and LUMO.

We first consider an Ag substrate and a positive bias larger than the LUMO energy, 0.9 eV. There is then a very efficient process in which a tip electron at an energy of about 0.9 eV, in a first step, tunnels from the tip to the LUMO. This is followed by another efficient process, in a second step, in which the LUMO electron tunnels to a substrate state at about 0.9 eV. A competing process to the second step is the tunneling of the HOMO electron to the substrate, creating a triplet exciton. This, however, leads to a configuration with an energy of at least 1.2-0.9=0.3 eV, depending on the energy of the substrate electron. In two following steps, the LUMO electron can tunnel to the substrate and a tip electron to the LUMO, creating a singlet exciton. This can be done in such a way that the whole process is over all (barely) energy conserving and allowed. However, the fact that there are intermediate configurations with finite positive energies means that this process is very inefficient compared with the first process. It is therefore unfavorable for up-conversion that the LUMO energy is smaller than the triplet energy.

The situation is similar for negative bias and an Au substrate. We consider a bias more negative than the HOMO energy at -1.1 eV. Then there is a very efficient sequence where the HOMO electron tunnels to a tip state at about -1.1 eV followed by the tunneling of a substrate electron at about -1.1 eV to the HOMO. The alternative of a substrate electron tunneling to the LUMO, in the second step, and creating a triplet exciton, leads to a configuration with an energy of at least 0.1 eV. This process is then inefficient. Similarly as above it is unfavorable that the absolute value of the HOMO energy is smaller than the triplet energy.

We now consider an Au substrate and positive bias. The LUMO energy is then larger than the triplet energy. The situation when $U_{\text{bias}} > E_{\text{LUMO}}$ leads to efficient up-conversion and has already been discussed in the literature.[29]

We therefore consider the case $E_{\text{Trip}} < U_{\text{bias}} < E_{\text{LUMO}}$ (Au substrate and up-conversion). In a first step (see Fig. 2 in the main text) a tip electron tunnels to the LUMO. The total energy is then

$$E_{\text{LUMO}} - E_T - U_{\text{bias}} > 0, \tag{11}$$

where $E_T < 0$ is the energy of the tip hole relative to the tip Fermi energy at $E_F^{\text{tip}} = U_{\text{bias}}$. The energy of the configuration in Eq. (11) is positive. In a second step a HOMO electron tunnels to a substrate level at the energy $E_S > 0$, creating a triplet exciton with the energy E_{Trip}. The total energy is

$$E_{\text{Trip}} - E_T - U_{\text{bias}} + E_S. \tag{12}$$

E_S and E_T can be chosen so that the energy of this configuration is zero. In a third step the LUMO electron tunnels to a substrate level at the energy $E_S^{'} > 0$. The total energy is

$$E_S + E_S^{'} - E_{\text{HOMO}} - E_T - U_{\text{bias}}. \tag{13}$$

In a fourth step a tip electron at the energy $U_{\text{bias}} + E_T^{'}$ ($E_T^{'} < 0$) tunnels to the LUMO, creating a singlet exciton. The total energy is

$$E_S + E_S^{'} - E_T - E_T^{'} - 2U_{\text{bias}} + E_{\text{sing}} = 0. \tag{14}$$

The energy of this final state must be the same as the energy of the initial state to make this four step process possible. We subtract Eq. (14) from Eq. (13) and obtain a new expression for the energy of the third step

$$E_T^{'} + U_{\text{bias}} - E_{\text{sing}} - E_{\text{HOMO}} < U_{\text{bias}} - E_{\text{sing}} - E_{\text{HOMO}} \tag{15}$$

where the inequality follows because $E_T^{'} < 0$. The right hand side is positive. There is then a

tip state $E_T^{'} < 0$ such that the energy in Eq. (13) is zero. This four step process then proceed via configurations for which only the second is not degenerate with the first and fifth. In contrast to the case of positive bias for an Ag substrate, there is no competing process proceeding entirely via degenerate configurations. This process is then, relatively, fairly efficient (although the choice of buffer plays a substantial role).

We next consider the case of up-conversion and negative bias for an Ag substrate, $U_{\text{bias}} < 0$ and $-E_{\text{Trip}} > U_{\text{bias}} > E_{\text{HOMO}}$. In a first step a HOMO electron tunnels to the tip at energy $U_{\text{bias}} + E_T$, with $E_T > 0$. The total energy

$$-E_{\text{HOMO}} + U_{\text{bias}} + E_T > 0. \tag{16}$$

is positive. In a second step a substrate electron at $E_S(< 0)$ tunnels to the LUMO and forms a triplet. The total energy is

$$E_{\text{Trip}} + U_{\text{bias}} + E_T - E_S, \tag{17}$$

which is zero for appropriate choices of E_T and E_S. In a third step the LUMO electron tunnels to tip at the energy $U_{\text{bias}} + E_T^{'}$, with $E_T^{'} > 0$. The total energy is

$$-E_S - E_{\text{HOMO}} + 2U_{\text{bias}} + E_T + E_T^{'}. \tag{18}$$

In the fourth step a substrate electron at $E_S^{'}$ tunnels to the LUMO. The total energy is

$$-E_S - E_S^{'} + E_T + E_T^{'} + 2U_{\text{bias}} + E_{\text{singlet}} = 0. \tag{19}$$

The final energy must be the same as the initial energy, so the energy in the fourth step is zero. We subtract the fourth step from the third step and obtain

$$E_S^{'} - E_{\text{HOMO}} - E_{\text{singlet}} < -E_{\text{HOMO}} - E_{\text{singlet}}. \tag{20}$$

For an Ag substrate the right hand side is positive. The energy $E_S^{'}$ can then be such that this expression is zero. The third step is then very efficient. This makes the whole four-step process

relatively efficient, competing with processes of comparable efficiency (but depending on the buffer).

References

(1) Grewal, A.; Leon, C. C.; Kuhnke, K.; Kern, K.; Gunnarsson, O. Character of Electronic States in the Transport Gap of Molecules on Surfaces. *ACS Nano* **2023**, *17*, 13176–13184.

(2) Grewal, A.; Leon, C. C.; Kuhnke, K.; Kern, K.; Gunnarsson, O. Scanning Tunneling Microscopy for Molecules: Effects of Electron Propagation into Vacuum. *ACS Nano* **2024**, *18*, 12158–12167.

(3) Harrison, W. *Elementary Electronic Structure*; WORLD SCIENTIFIC, 1999.

(4) Leon, C. C.; Grewal, A.; Kuhnke, K.; Kern, K.; Gunnarsson, O. Anionic Character of the Conduction Band of Sodium Chloride. *Nat. Commun.* **2022**, *13*, 981.

(5) Davey, W. P. Precision Measurements of the Lattice Constants of Twelve Common Metals. *Phys. Rev.* **1925**, *25*, 753–761.

(6) Chen, H.-Y. T.; Pacchioni, G. Properties of Two-Dimensional Insulators: A DFT Study of Co Adsorption on NaCl and MgO Ultrathin Films. *Phys. Chem. Chem. Phys.* **2014**, *16*, 21838–21845.

(7) Roth, F.; Lupulescu, C.; Darlatt, E.; Gottwald, A.; Eberhardt, W. Angle Resolved Photoemission from Cu Single Crystals: Known Facts and a Few Surprises about the Photoemission Process. *J. Electron Spectrosc. Relat. Phenom.* **2016**, *208*, 2–10.

(8) Roth, F.; Arion, T.; Kaser, H.; Gottwald, A.; Eberhardt, W. Angle Resolved Photoemission from Ag and Au Single Crystals: Final State Lifetimes in the Attosecond Range. *J. Electron Spectrosc. Relat. Phenom.* **2018**, *224*, 84–92.

(9) Sheverdyaeva, P. M.; Requist, R.; Moras, P.; Mahatha, S. K.; Papagno, M.; Ferrari, L.; Tosatti, E.; Carbone, C. Energy-Momentum Mapping of d -Derived Au(111) States in a Thin Film. *Phys. Rev. B* **2016**, *93*, 035113.

(10) Sirdeshmukh, D. B.; Sirdeshmukh, L.; Subhadra, K. G. *Alkali Halides: A Handbook of Physical Properties*; Springer Series in Materials Science; Springer: Berlin, Heidelberg, 2001; p 6.

(11) Schulzendorf, M.; Hinaut, A.; Kisiel, M.; Jöhr, R.; Pawlak, R.; Restuccia, P.; Meyer, E.; Righi, M. C.; Glatzel, T. Altering the Properties of Graphene on Cu(111) by Intercalation of Potassium Bromide. *ACS Nano* **2019**, *13*, 5485–5492.

(12) Shannon, R. D. Revised Effective Ionic Radii and Systematic Studies of Interatomic Distances in Halides and Chalcogenides. *Acta Cryst. A* **1976**, *32*, 751–767.

(13) Roessler, D. M.; Walker, W. C. Electronic Spectrum of Crystalline Lithium Fluoride. *J. Phys. Chem. Solids* **1967**, *28*, 1507–1515.

(14) Watkins, N. J.; Gao, Y. Vacuum Level Alignment of Pentacene on LiF/Au. *J. Appl. Phys.* **2003**, *94*, 1289–1291.

(15) Schintke, S.; Schneider, W.-D. Insulators at the Ultrathin Limit: Electronic Structure Studied by Scanning Tunnelling Microscopy and Scanning Tunnelling Spectroscopy. *J. Phys.: Condens. Matter* **2004**, *16*, R49–R81.

(16) Heo, S.; Cho, E.; Lee, H.-I.; Park, G. S.; Kang, H. J.; Nagatomi, T.; Choi, P.; Choi, B.-D. Band Gap and Defect States of MgO Thin Films Investigated Using Reflection Electron Energy Loss Spectroscopy. *AIP Adv.* **2015**, *5*, 077167.

(17) Wasada-Tsutsui, Y.; Tatewaki, H. Electronic Band Structure of Crystalline NaF: Ionization Threshold and Excited States Related to Lattice Defects. *Surf. Sci.* **2002**, *513*, 127–139.

(18) Poole, R. T.; Jenkin, J. G.; Liesegang, J.; Leckey, R. C. G. Electronic Band Structure of the Alkali Halides. I. Experimental Parameters. *Phys. Rev. B* **1975**, *11*, 5179–5189.

(19) Hedin, L. New Method for Calculating the One-Particle Green's Function with Application to the Electron-Gas Problem. *Phys. Rev.* **1965**, *139*, A796–A823.

(20) Wang, S.; Kharche, N.; Costa Girão, E.; Feng, X.; Müllen, K.; Meunier, V.; Fasel, R.; Ruffieux, P. Quantum Dots in Graphene Nanoribbons. *Nano Lett.* **2017**, *17*, 4277–4283.

(21) KBr Crystal Structure - SpringerMaterials. https://materials.springer.com/isp/crystallographic/docs/sd_1711016, Last accessed: 13.02.2024.

(22) Wells, A. F. *Structural Inorganic Chemistry*, 5th ed.; Clarendon Press, 1984.

(23) Arveson, S. M.; Kiefer, B.; Deng, J.; Liu, Z.; Lee, K. K. M. Thermally Induced Coloration of KBr at High Pressures. *Phys. Rev. B* **2018**, *97*, 094103.

(24) RbI Crystal Structure - SpringerMaterials. https://materials.springer.com/isp/crystallographic/docs/sd_0545585, Last accessed: 13.02.2024.

(25) Rubidium Iodide. https://www.ucl.ac.uk/~ucapahh/research/crystal/rbi.htm, Last accessed: 13.02.2024.

(26) Miwa, K.; Imada, H.; Kawahara, S.; Kim, Y. Effects of Molecule-Insulator Interaction on Geometric Property of a Single Phthalocyanine Molecule Adsorbed on an Ultrathin NaCl Film. *Phys. Rev. B* **2016**, *93*, 165419.

(27) Li, Z.; Chen, H.-Y. T.; Schouteden, K.; Janssens, E.; Haesendonck, C. V.; Lievens, P.; Pacchioni, G. Spontaneous Doping of Two-Dimensional NaCl Films with Cr Atoms: Aggregation and Electronic Structure. *Nanoscale* **2015**, *7*, 2366–2373.

(28) Chen, G.; Luo, Y.; Gao, H.; Jiang, J.; Yu, Y.; Zhang, L.; Zhang, Y.; Li, X.; Zhang, Z.; Dong, Z. Spin-Triplet-Mediated Up-Conversion and Crossover Behavior in Single-Molecule Electroluminescence. *Phys. Rev. Lett.* **2019**, *122*, 177401.

(29) Luo, Y.; Kong, F.-F.; Tian, X.-J.; Yu, Y.-J.; Jing, S.-H.; Zhang, C.; Chen, G.; Zhang, Y.; Zhang, Y.; Li, X.-G.; Zhang, Z.-Y.; Dong, Z.-C. Anomalously bright single-molecule upconversion electroluminescence. *Nature Communications* **2024**, *15*, 1677.
